# Supplementary material for: Stimuli-Sensitive Platinum-Based Anticancer Polymer Therapeutics: Synthesis and Evaluation In Vitro
Source: Pharmaceutics. 2025 Nov 5;17(11):1433. doi: 10.3390/pharmaceutics17111433 (PMC12655766; doi:10.3390/pharmaceutics17111433)
Supplement: Supplementary file 1 [file pharmaceutics-17-01433-s001.zip › pharmaceutics-3930742-supplementary.pdf]

pharmaceutics

MDPI

Supplementary information

Stimuli-Sensitive Platinum-Based Anticancer Polymer Therapeutics: Synthesis and Evaluation In Vitro

Kateřina Běhalová [1], Martin Studenovský [2,*], Kevin Kotalík [2], Rafal Konefal [2,3], Marek Kovář [1] and Tomáš Etrych [2]

[1] Institute of Microbiology of the Czech Academy of Sciences, v.v.i., Vídeňská 1083, 14220 Prague, Czech Republic; katerina.behalova@biomed.cas.cz (K.B.); makovar@biomed.cas.cz (M.K.)
[2] Institute of Macromolecular Chemistry of the Czech Academy of Sciences, v.v.i., Heyrovského sq. 2, 16206 Prague, Czech Republic; kotalik@imc.cas.cz (K.K.); rafal.konefal@amu.edu.pl (R.K.); etrych@imc.cas.cz (T.E.)
[3] NanoBioMedical Centre, Adam Mickiewicz University, Wszechnicy Piastowskiej 3, 61-614 Poznan, Poland
* Correspondence: studenovsky@imc.cas.cz; Tel.: +42-029-680-9230

Academic Editor: Donato Cosco

Received: 29 September 2025
Revised: 31 October 2025
Accepted: 3 November 2025
Published: 5 November 2025

Citation: Běhalová, K.; Studenovský, M.; Kotalík, K.; Konefal, R.; Kovář, M.; Etrych, T. Stimuli-Sensitive Platinum-Based Anticancer Polymer Therapeutics: Synthesis and Evaluation In Vitro. *Pharmaceutics* **2025**, *17*, 1433. https://doi.org/10.3390/pharmaceutics17111433

Copyright: © 2025 by the authors. Licensee MDPI, Basel, Switzerland. This article is an open access article distributed under the terms and conditions of the Creative Commons Attribution (CC BY) license (https://creativecommons.org/licenses/by/4.0/).

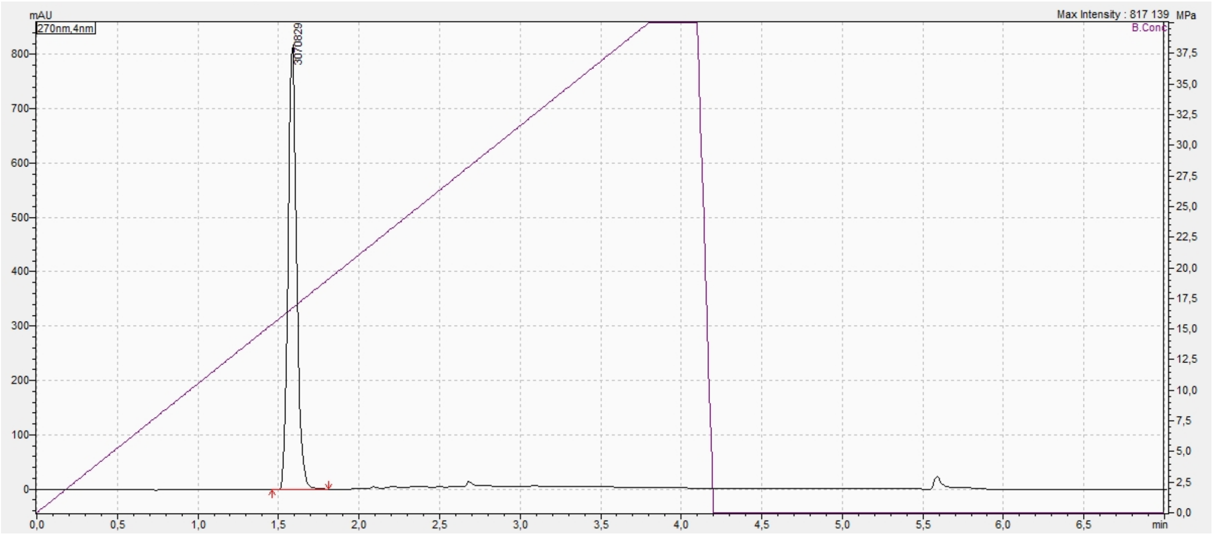

Figure S1. Calibration HPLC chromatogram of Pt-OBP: The peak at 1.6 min corresponds to the 20 μL injection of 1 mg/mL solution of Pt-OBP in methanol. A mixture of water–acetonitrile in the presence of 0.1% TFA was used as the eluent at a gradient 0–100 vol % and a flow rate of 4 mL/min. The acetonitrile concentration gradient (with 0.1% TFA) is indicated in purple in the chromatogram.

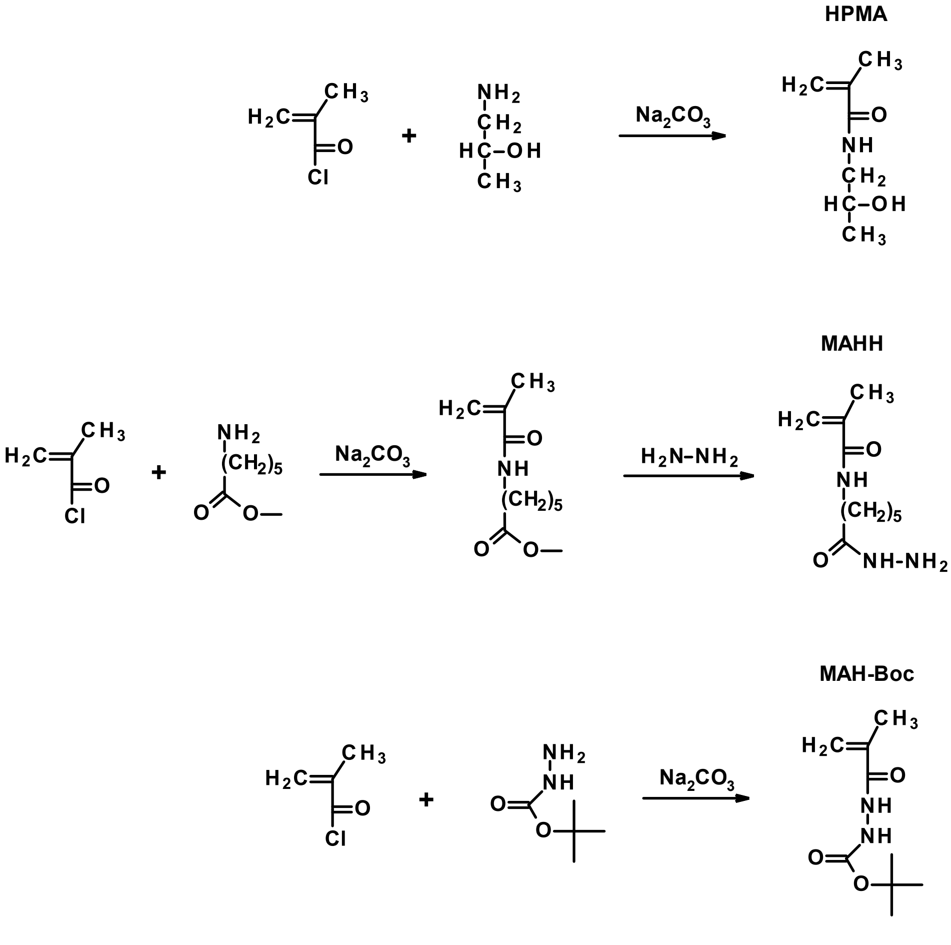

Figure S2. Scheme of synthesis of monomers HPMA, MAHH and MAH-Boc.

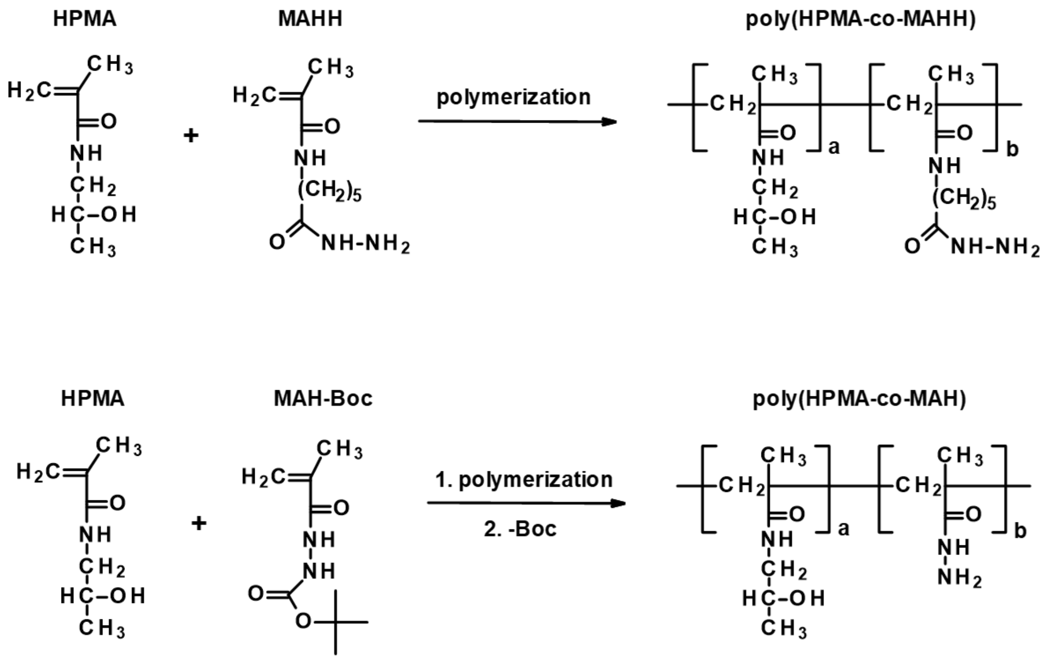

Figure S3. Scheme of synthesis of polymer precursors based on MAHH and MAH-Boc comonomers.

Disclaimer/Publisher's Note: The statements, opinions and data contained in all publications are solely those of the individual author(s) and contributor(s) and not of MDPI and/or the editor(s). MDPI and/or the editor(s) disclaim responsibility for any injury to people or property resulting from any ideas, methods, instructions or products referred to in the content.
